# Sickle cell disease and pregnancy outcomes: a study of the community-based hospital in a tribal block of Gujarat, India

**DOI:** 10.1186/s41043-017-0079-z

**Published:** 2017-01-21

**Authors:** Gayatri Desai, Ankit Anand, Pankaj Shah, Shobha Shah, Kapilkumar Dave, Hardik Bhatt, Shrey Desai, Dhiren Modi

**Affiliations:** 1 Kasturba Maternity Hospital, SEWA Rural, Bharuch, Gujarat India; 2 Community Health Project, SEWA Rural, Bharuch, Gujarat India; 3 Population Research Centre, Institute for Social and Economic Change, Bangalore, India; 4 Women’s Health and Training, SEWA Rural, Bharuch, Gujarat India

**Keywords:** Sickle cell disease, Pregnancy outcomes, Maternal health, Tribal, Gujarat

## Abstract

**Background:**

Sickle cell disease (SCD) is a hereditary blood disorder prevalent in tribal regions of India. SCD can increase complications during pregnancy and in turn negatively influence pregnancy outcomes. This study reports the analysis of tribal maternal admissions in the community-based hospital of SEWA Rural (Kasturba Maternity Hospital) in Jhagadia block, Gujarat. The objective of the study is to compare the pregnancy outcomes among SCD, sickle cell trait and non-SCD admissions. This study also estimated the risk of adverse pregnancy outcomes for SCD admissions.

**Methods:**

The data pertains to four and half years from March 2011 to September 2015. The total tribal maternal admissions were 14640, out of which 10519 admissions were deliveries. The admissions were classified as sickle cell disease, sickle cell trait and non-sickle cell disease. The selected pregnancy outcomes and maternal complications were abortion, stillbirth, Caesarean section, haemoglobin levels, blood transfusion, preterm pregnancy, newborn birth weight and other diagnosed morbidities (IUGR, PIH, eclampsia, preterm labour pain). The odds ratios for each risk factor were estimated for sickle cell patients. The odds ratios were adjusted for the respective years.

**Results:**

Overall, 1.2% (131 out of 10519) of tribal delivery admissions was sickle cell admissions. Another 15.6% (1645 out of 10519) of tribal delivery admissions have sickle cell trait. The percentage of stillbirth was 9.9% among sickle cell delivery admission compared to 4.2% among non-sickle cell deliveries admissions. Among sickle cell deliveries, 70.2% were low birth weight compared to 43.8% of non-sickle cell patient. Similarly, almost half of the sickle cell deliveries needed the blood transfusion. The 45.0% of sickle cell delivery admissions were pre-term births, compared to 17.3% in non-SCD deliveries. The odds ratio of severe anaemia, stillbirth, blood transfusion, Caesarean section, and low birth weight was significantly higher for sickle cell admissions compared to non-sickle cell admissions.

**Conclusions:**

The study exhibited that there is a high risk of adverse pregnancy outcomes for women with SCD. It may also be associated with the poor maternal and neonatal health in these tribal regions. Thus, the study advocates the need for better management of SCD in tribal Gujarat.

## Background

Sickle cell disease (SCD) is a hereditary blood disorder, prevalent in sub-Saharan Africa, South America, Central America, Saudi Arabia, India, and Mediterranean countries [1, 2]. It is the most common inherited condition worldwide [2]. Estimates showed the trend of increasing number of people with SCD, mostly from developing countries [3, 4]. Globally, India accounts for 14.5% of the total newborns with SCD [4]. SCD can increase complication during pregnancy and in turn negatively influence the pregnancy outcomes [4–6]. Studies in Africa and United Kingdom (UK) have tried to estimate the negative influence of SCD on pregnancy outcomes. Studies on SCD pregnancy have focused on the risks to the foetus, including preterm labour and intrauterine growth retardation (IUGR) [7, 8]. The majority of studies have shown that SCD is negatively associated with maternal health and perinatal conditions [7–9]. Pregnant women with SCD have increased chances of pregnancy-related complications and infections [8, 9]. There are not many studies in India, which have explored the risk of negative pregnancy outcomes with SCD [10, 11]. The Indian studies on SCD were of small sample sizes and had issue with the generalization of their results [12–15]. SCD is prevalent in the tribal population of Odisha, Gujarat, Madhya Pradesh, Chhattisgarh and Rajasthan [4, 10, 16, 17]. In Gujarat, it is estimated that 1–2 million tribals have sickle cell trait and approximately 80,000 people are affected by SCD [18]. This calls for understanding the associated risk for pregnant women with SCD in tribal regions of Gujarat. This study reports the analysis of the tribal maternal admissions, in the community-based hospital of SEWA Rural (Kasturba Maternity Hospital) in Jhagadia block, Gujarat. The objective of the study is to compare the pregnancy outcomes among SCD, sickle cell trait and non-SCD maternal admissions. The study has also estimated the risk of adverse pregnancy outcomes for women with SCD.

## Methods

### Study setting

We used the data of all maternal admissions in the Kasturba Maternity Hospital (Hospital of SEWA Rural NGO). The hospital functions in Jhagadia block. Jhagadia block consists of 164 villages with a population of 185,000 [19]. Around 70% of the population of Jhagadia is tribal [19]. The Kasturba Maternity Hospital has been providing maternal and neonatal health services in this area for the last 35 years. The hospital works as a first referral unit and the biggest provider of maternal health care in the Jhagadia block.

### Data sources and sample size

**Fig. 1:**
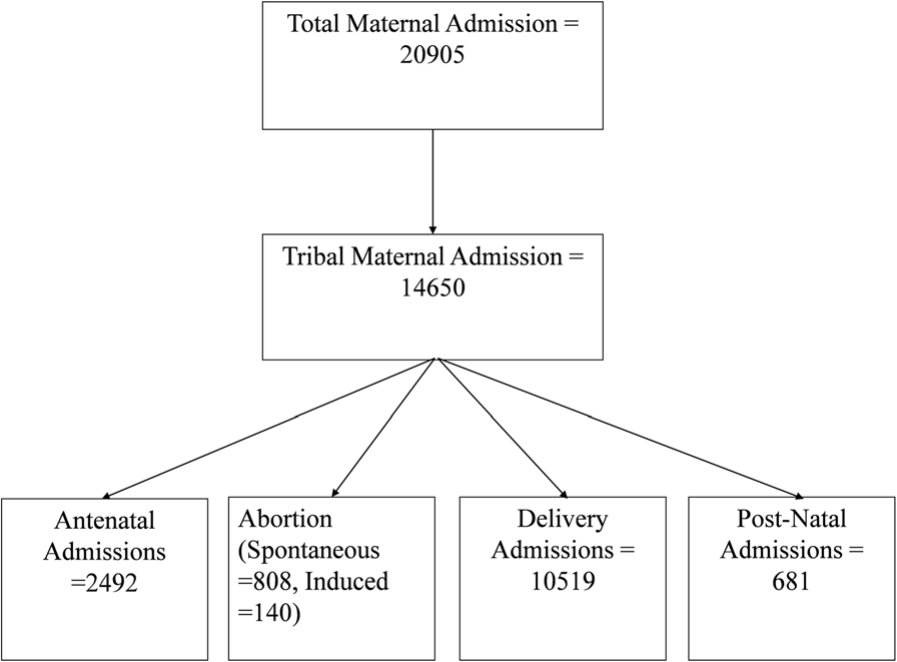
Description of all maternal admission in Kasturba Maternity Hospital (2011–2015), Jhagadia, Gujarat

The hospital keeps a regularly updated register for every enrolled pregnant woman. The register has been maintained since 2003. The data pertain to four and half years from March 2011 to September 2015; SCD information was not widely collected before March 2011. The data entry operators enter the hospital register information every day. A gynaecologist used to check for discrepancies in the data on a weekly basis. SCD (haemoglobin SS disease) can only occur to tribal population because it requires sickle cell genotype from both parents. We define women as tribal according to the listing of the government of India [20]. The total maternal admissions during that period were 20,950, out of which 14,650 were tribal maternal admissions (Fig. 1). Maternal admissions include abortion (both induced and spontaneous), antenatal care, delivery and postnatal care. We were interested in pregnancy outcomes, so we have taken only delivery admissions as the final sample. The final sample size and total tribal delivery admission was 10,519 (Fig. 1).

### Measurement and classification of the variables

All the admissions were classified as SCD, sickle cell trait and non-SCD admissions. The sickle cell disease genotype (haemoglobin SS disease) was defined as SCD [21]. Other forms of sickle cell (where sickle cell comes only from one parent) were categorized as sickle cell trait [21]. Information about maternal outcomes such as pregnancy results, diagnosis and procedure performed were recorded for each of the admission. We have used the information on the pregnancy outcomes, such as abortion, live/stillbirth, birth weight of the children, gestational week and haemoglobin level of the mother. The classification of variables is given in Table 1. Low birth weight was defined as weight less than 2.5 kg and severe low birth weight was defined as weight less than 1.5 kg. Anaemia among pregnant women defined as haemoglobin level less than 11 g/dl, and severe anaemia defined as haemoglobin level less than 7 g/dl. The hospital uses Hemocue 301 to detect the haemoglobin levels. Birth before 36 gestational weeks was labelled as pre-term pregnancy. Procedures such as Caesarean section, blood transfusion, and diagnosed morbidities (sickle cell crises, IUGR, PIH, eclampsia, abruption, and Preterm Labour Pain) were also analysed.

**Table 1:** Description and classification of the variables

Variables	Classification
Sickle cell disease	Sickle cell disease (SCD)
	Sickle cell trait
	No sickle cell disease/trait
Pregnancy results	Spontaneous abortion
	Induced abortion
	Live birth
	Stillbirth
Haemoglobin level of the mother	Severe anaemia (HB ≤7g/dl)
	Anaemia (HB ≤11g/dl)
Gestational week	Pre-term (≤36 gestational week)
	Full-term (37–42 weeks)
	Post-term (>42 weeks)
Blood transfusion	0
	1–2
	3 and more
Caesarean section (for live/stillbirth)	Yes
	No
Birth weight of the children (for live/stillbirth)	Severely low birth weight (<1.5 kg)
	Low birth weight (<2.5 kg)
	Normal birth weight (>2.5 kg)
Diagnose morbidities during pregnancy	
• Sickle cell crises (only for sickle cell patient)	Yes, No
• Intrauterine growth retardation	Yes, No
• Pregnancy-induced hypertension	Yes, No
• Eclampsia	Yes, No
• Preterm labour pain	Yes, No

### Statistical analysis

Cross tabulation was done to calculate the pregnancy outcomes, diagnosis morbidities and treatment procedures by sickle cell disease groups. Missing values were excluded from the analysis. Logistic regression was performed to compute the risk of these pregnancy outcomes. Each of the pregnancy outcomes and conditions was taken as a dependent variable and SCD status as an independent variable. Non-sickle cell admission was compared to SCD admission and sickle cell trait admissions. Odds ratios were also estimated comparing sickle cell trait with SCD admissions. All of the odds ratios were adjusted for the year of the admissions. All analyses were performed using STATA version 12.0 software [22].

## Results

**Fig. 2:**
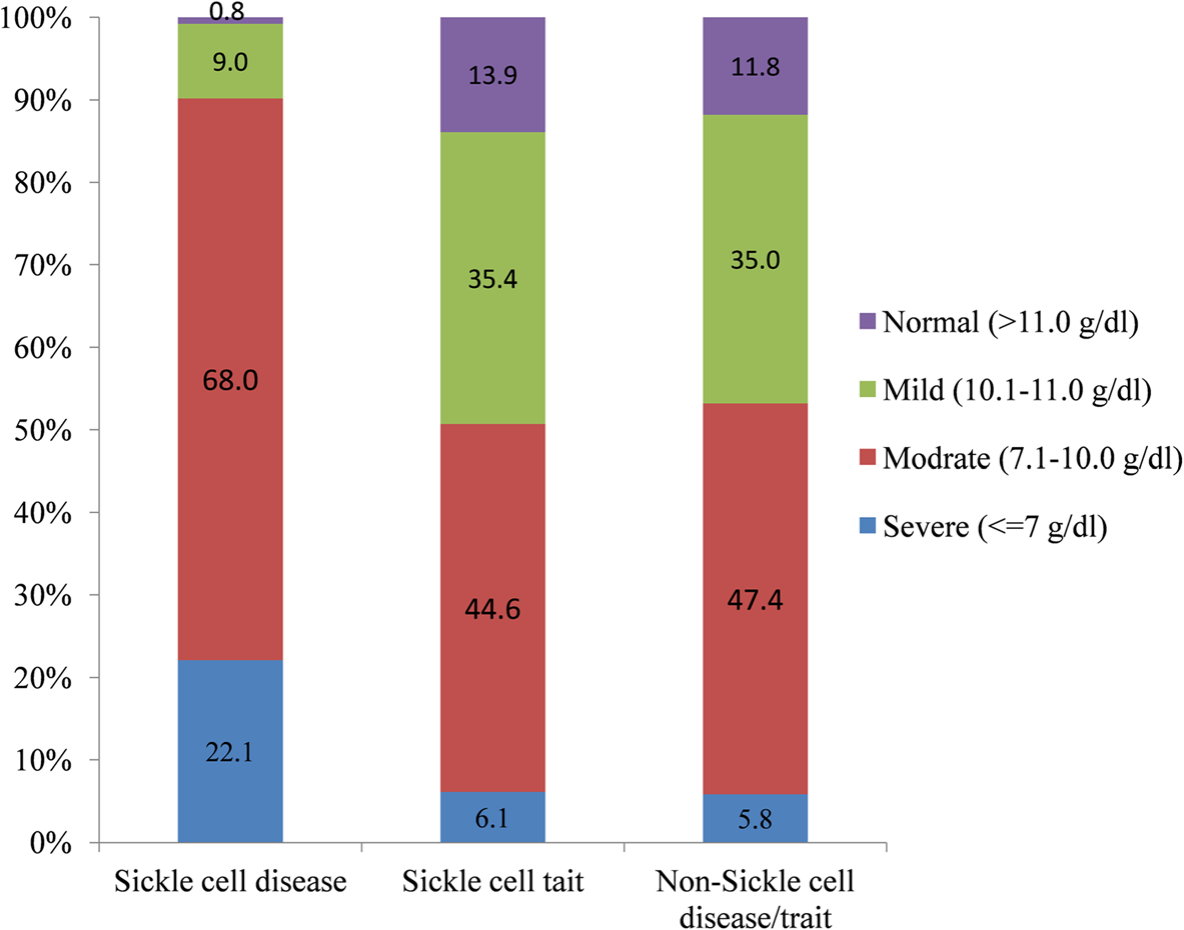
Haematological (haemoglobin status) profile of all admission by sickle cell status

Maternal admissions by SCD status are demonstrated in Table 2. The percentage of sickle cell admissions was 1.2% (131 out of 10519) of tribal delivery admissions, and all of the women had homozygous sickle cell disease genotype (HBSS). The percentage of sickle cell admissions were 1.2% (131 out of 10,519) of tribal delivery admissions. Another 15.6% (1645 out of 10,519) of tribal delivery admissions have the sickle cell trait. The number of abortions both spontaneous and induced was quite small. Not much can be inferred about the relationship between SCD and abortion. The missing values in SCD status have declined over the years. The SCD status was unknown for around 4.6% of overall maternal admissions. More than 99% of SCD admissions were anaemic (Fig. 2). Among sickle cell trait and non-SCD admissions, 86.1 and 88.2% of admissions were anaemic. This indicates that there is a very high prevalence of anaemia among pregnant women in this region. The percentage of severely anaemic admission was 6.1 and 5.8% among sickle cell trait and non-SCD admissions, respectively, compared to 22.1% among SCD admissions.

**Table 2:** Number and percentages of maternal admission by SCD status

		SCD *N* (%)	SCD trait *N* (%)	Non-SCD *N* (%)	Not available *N* (%)
Delivery	2011–2012	26 (1.3)	262 (13.4)	1400 (71.9)	260 (13.3)
	2012–2013	25 (1.2)	290 (13.7)	1641 (77.5)	162 (7.6)
	2013–2014	30 (1.3)	386 (16.1)	1920 (80.2)	57 (2.4)
	2014–2015	30 (1.2)	430 (17.2)	2040 (81.5)	2 (0.1)
	2015–2016	20 (1.3)	277 (17.8)	1259 (80.9)	1 (0.1)
	Total	131 (1.2)	1645 (15.6)	8260 (78.5)	482 (4.6)
Spontaneous abortion	2011–2012	2 (1.3)	16 (10.5)	105 (68.6)	30 (19.6)
	2012–2013	1 (0.6)	27 (17.0)	110 (69.2)	21 (13.2)
	2013–2014	4 (2.3)	30 (17.0)	133 (75.6)	9 (5.1)
	2014–2015	5 (2.3)	41 (19.2)	166 (77.9)	1 (0.5)
	2015–2016	0 (0.0)	18 (17.0)	86 (81.1)	2 (1.9)
	Total	12 (1.5)	132 (16.4)	600 (74.3)	63 (7.8)
Induced abortion	2011–2012	1 (3.3)	4 (13.3)	19 (63.3)	6 (20)
	2012–2013	0 (0)	3 (10.7)	20 (71.4)	5 (17.9)
	2013–2014	0 (0)	4 (15.4)	20 (76.9)	2 (7.7)
	2014–2015	1 (2.3)	11 (25.6)	31 (72.1)	0 (0)
	2015–2016	0 (0.0)	0 (0.0)	13 (100)	0 (0)
	Total	2 (1.4)	22 (15.7)	103 (73.6)	13 (9.3)

Table 3 presents the relationship between pregnancy outcomes and SCD. Among SCD admissions, 9.9% resulted in stillbirth compared to 4.4% in the trait of the disease and 3.6% in non-SCD admissions. More than half of SCD admissions (52.7%) needed the blood transfusion, and 8.4% of SCD admissions had three or more blood transfusions. Almost 43% of deliveries were resulted in low birth weight among non-SCD admission, and 4.2% of non sickle cell deliveries were resulted in severely low birth weight. Among SCD deliveries, almost 70% (two out of three) were low birth weight. The percentage of severely low birth weight among SCD deliveries was 8.4%. Less than half (45.6%) of the SCD deliveries were also preterm, compared to 17.0% in non-SCD deliveries. Half of the SCD admission had the pain crisis during pregnancy. Table 4 shows the risk (odds ratio) of negative pregnancy outcome in SCD comparing with non-SCD and sickle cell trait admissions. The odds ratio of stillbirth among SCD was three times higher compared to non-SCD patients. The odds ratio of low birth weight for SCD deliveries was three times higher compared to non-SCD admissions. The odds ratio of severely low birth weight was two times higher in SCD deliveries compared to non-SCD deliveries. The risk (odds ratio) of pre-term delivery and Caesarean section is more than three times higher among SCD admissions compared to non-SCD admissions. The risk was similar and non-significant between sickle cell trait and non-SCD admissions. The stillbirth and blood transfusion rate was significantly higher for sickle cell trait compared to non-SCD admissions. The risk of adverse pregnancy outcomes was significantly higher among SCD admission, in comparison with sickle cell trait and non-SCD admissions.

**Table 3:** Pregnancy outcomes and maternal complications by SCD status of women who delivered in the hospital

		SCD *N* (%)	Sickle cell trait *N* (%)	Non-SCD *N* (%)
Type of birth	Live birth	118 (90.1)	1573 (95.6)	8001 (96.9)
	Stillbirth	13 (9.9)	72 (4.4)	258 (3.1)
Blood transfusion	No	62 (47.3)	1575 (95.8)	7998 (97.1)
	Yes	69 (52.7)	69 (4.2)	239 (2.9)
Number of blood transfusion	0	62 (47.3)	1575 (95.8)	7998 (97.1)
	1–2	58 (44.3)	67 (4.1)	221 (2.7)
	3 and more	11 (8.4)	2 (0.1)	18 (0.2)
Birth weight	≤1499 g	11 (8.4)	76 (4.6)	351 (4.2)
	1500–1999 g	19 (14.5)	125 (7.6)	648 (7.8)
	2000–2499 g	62 (47.3)	520 (31.6)	2627 (31.8)
	≥2500 g	39 (29.8)	924 (56.2)	4633 (56.1)
Gestational weeks	Pre-term (≤36 weeks)	59 (45.0)	287 (17.4)	1466 (17.7)
	Full-term (37–42 weeks)	72 (55.0)	1355 (82.4)	6789 (82.2)
	Post-term (>42 weeks)	0 (0.0)	3 (0.2)	5 (0.1)
Caesarean section	No	108 (82.4)	1553 (94.4)	7754 (93.9)
	Yes	23 (17.6)	92 (5.6)	506 (6.1)
Sickle crisis	No	69 (52.7)	NA	NA
	Yes	62 (47.3)	NA	NA
Intrauterine growth retardation	No	128 (97.7)	1614 (98.1)	8126 (98.4)
	Yes	3 (2.3)	31 (1.9)	134 (1.6)
Pregnancy-induced hypertension	No	123 (93.9)	1509 (91.7)	7578 (91.7)
	Yes	8 (6.1)	136 (8.3)	682 (8.3)
Eclampsia	No	128 (97.7)	1628 (99)	8172 (98.9)
	Yes	3 (2.3)	17 (1)	88 (1.1)
Preterm labour pain	No	130 (99.2)	1643 (99.9)	8254 (99.9)
	Yes	1 (0.8)	2 (0.1)	6 (0.1)

*NA* not applicable

**Table 4:** Risk (odds ratio with 95% CI) of adverse pregnancy outcomes and maternal complication by sickle cell disease status

	Negative (Ref) vs. SCD	Negative (Ref) vs. sickle cell trait	Sickle cell trait (Ref) vs. SCD
Spontaneous abortion	0.78 (0.43–1.39)	1.09 (0.78–1.22)	0.71 (0.39–1.30)
Stillbirth	3.45 (1.92–6.21)**	1.41 (1.09–1.85)*	2.43 (1.31–4.53)**
Blood transfusion	37.66 (26.08–54.39)**	1.46 (1.11–1.91)**	25.88 (16.99–39.41)**
Severe anaemia	1.28 (0.87–1.88)	1.05 (0.93–1.41)	1.22 (0.82–1.82)
Any anaemic	13.84 (1.93–99.51)**	0.85 (0.71–1.00)	16.39 (2.28–117.92)**
Severely low birth weight ≤1499	2.07 (1.11–3.87)*	1.09 (0.85–1.41)	1.89 (0.98–3.66)
Low birth weight ≤2499	3.01 (2.06–4.39)**	1.01 (0.90–1.11)	2.99 (2.03–4.41)**
Pre-term delivery (gestational weeks ≤36 weeks)	3.88 (2.73–5.51)**	0.98 (0.85–1.13)	3.96 (2.74–5.72)**
Caesarean section	3.79 (2.31–6.21)**	0.86 (0.68–1.08)	4.42 (2.60–7.52)
Intrauterine growth retardation (IUGR)	1.44 (0.45–5.46)	1.15 (0.78–1.71)	1.25 (0.38–4.14)
Pregnancy-induced hypertension (PIH)	0.73 (0.36–1.51)	0.99 (0.82–1.20)	0.74 (0.35–1.55)
Eclampsia	2.18 (0.68–6.97)	0.98 (0.58–1.65)	2.22 (0.64–7.69)

*Ref* reference category

*P < 0.05; **P < 0.01

## Discussion

The study compared the pregnancy outcomes between SCD and non-SCD pregnant women. The results of our study were consistent with previous studies in different countries. Similar to our results, studies in Africa and the United Kingdom have estimated around 1% of all pregnancies had SCD [23, 24]. The high prevalence of anaemia among women and the presence of SCD has remained a hurdle for improving maternal health in India [25]. Our study showed that women with the SCD have higher chances of stillbirth, low birth weight and pre-term birth compared to the SCD trait and non-SCD pregnancies. Studies in African and European countries have reported similar findings; the SCD deliveries have higher chance of lower birth weights, low gestational period and increased stillbirth rate compared to the non-SCD deliveries [7, 23, 26, 27]. SCD can also lead to pain crisis and cause mortality [12, 14]. Around half of the pregnant SCD women have sickle cell crisis in our sample. Mutiple studies have reported high risk of abortion among SCD women [7–9, 23, 26]. In our study, the number of abortions was quite small and the association with SCD could not be established. We also did not find any significant relationship between diagnosed morbidities and SCD as depicted in previous studies [28–30], the reason may be the less number of women with morbidities in our sample.

The sickling of red blood cells in SCD can contribute to micro-vascular damage [31]. These physiological changes due to SCD may lead to complication and affect foetal growth during pregnancy [32]. There are no clear strategies to improve maternal outcomes for women with SCD in low resource settings [30]. In high-income countries, pregnant women with SCD are managed by a group of specialist such as obstetrician, haematologist and trained midwives. The specialized care may not be available in remote tribal and rural areas of India [33]. The need for scrupulous health care for pregnant women and newborn with SCD is crucial in improving maternal and neonatal health in the tribal areas [34]. Identification of women with higher risk of adverse outcomes, regular third trimester screening for foetal growth and blood transfusion for women with SCD may represent a strategy to reduce adverse maternal outcomes [33–35]. There is a high need to research on effective interventions to reduce negative maternal complication among women with SCD in low-middle income countries [30].

There are some limitations of this study. This study is based on data collected at a hospital and does not contain information regarding women with SCD who delivered at home. Therefore, one might need to be cautious before generalizing findings of the study to those women who delivered at home. The study is based on maternal admissions; some women may have more than one admission during the period of study.

## Conclusions

This is one of the few studies in India, which have estimated pregnancy outcomes for women with SCD. The study exhibited that there is a high risk of adverse pregnancy outcomes for women with SCD compared to non-SCD and sickle cell trait admissions. The presence of SCD combined with anaemia among tribal women in Gujarat is the biggest challenge in achieving better maternal health in this region. Thus, the study advocates the need for better management of SCD and anaemia in the tribal regions of Gujarat.

## Abbreviations

BT: Blood transfusion
IUGR: Intrauterine growth retardation
PIH: Pregnancy-induced hypertension
SCD: Sickle cell disease
